# Reply to Pawar et al

**DOI:** 10.1093/infdis/jiu034

**Published:** 2014-01-22

**Authors:** Stacy Todd, Erwin De Bruin, Nguyen Thi Duy Nhat, Marion Koopmans, Maciej F. Boni

**Affiliations:** 1 Oxford University Clinical Research Unit, Wellcome Trust Major Overseas Programme, Ho Chi Minh City, Vietnam; 2 Liverpool School of Tropical Medicine, Liverpool; 3 Centre for Tropical Medicine, Nuffield Department of Clinical Medicine, University of Oxford, Oxford, United Kingdom; 4 National Institute for Public Health and the Environment, Bilthoven; 5 Department of Virology, Erasmus Medical Center, Rotterdam, the Netherlands

To the Editor—In this issue of the *Journal* and in a recent study, Pawar et al reported results of serologic tests performed on a high-risk group of 446 poultry workers and 162 individuals from the general population in Maharashtra and Jamshedpur states, India [1, 2]. None of the 608 samples tested positive for antibodies to influenza A virus subtypes H5N1 or H7N1 by hemagglutination-inhibition (HI) or microneutralization (MN) assays. None of the 162 individuals from the general population tested positive for H9N2 antibodies by HI or MN assay. A total of 4.7% and 10% of high-risk individuals in Pune and Jamshedpur, respectively, tested positive by the HI assay for influenza A(H9N2); 3.8% and 4.7%, respectively, had positive results of the MN assay. The authors suggest that this higher rate of seropositivity could be related to the circulation of influenza A(H9N2) in Jamshedpur in poultry, with resultant zoonotic spread to humans, and reference our publication showing presence of antibodies to avian influenza virus antigens in Vietnam.

As noted by the authors, H9 titers were highest among all antibodies to avian influenza virus strains in our general population sample [3]. Other publications looking at high-risk individuals in South East Asia have demonstrated a higher seropositivity rate for influenza A(H9) strains, compared with influenza A(H5) and/or A(H7) strains [4–6], but this is not a consistent finding globally, even in high-risk groups [7, 8]. Vaccine studies conducted in locations thought to have a low risk of avian influenza exposure (ie, the United Kingdom and United States) found that up to a third of participants were seropositive for H9 [9–11]. Of those positive for H9 antibodies (>1:40 by the HI assay), a greater proportion were born before 1968, and it was postulated that this was related to cross-reactivity from influenza A virus subtype H2N2, which was circulating in humans from 1957 to 1968. Within our data set, there was a significantly higher seropositivity (and, accordingly, mean titer) for all avian strains in individuals born before 1968 (multivariate analysis of variance [MANOVA]: F = 4.7, *P* < .0005, Pillai 0.1256), with the H9 titer being the highest. However, when we investigate other birth year cutoffs (±20 years), we find the same trend, suggesting that this effect is more related to an increase in age rather than to a specific exposure event.

In the Vietnam sample set (n = 1424; slightly reduced due to quality checks from our original sample set), antibody titers to avian influenza A virus antigens increased with age. The optimal fitted regression curve among nonlinear models was a fifth-order polynomial (ANOVA: *P* < .001), but this curve provided no additional qualitative explanations of the data than the second best fit model, a simple quadratic regression (Figure 1). Titers to H9 antigen increase more rapidly with age than titers to H7 or H5. One hypothesis is that this difference is caused by varying levels of exposure to avian influenza viruses. A second hypothesis is that this is caused by differences in cross-reactivity between human influenza antibodies and each of the subtypes H9, H7, and H5. Our analysis provides more support for the second hypothesis (supplementary figures 1 and 2 in the article by Boni et al [3]).

**Figure 1.**
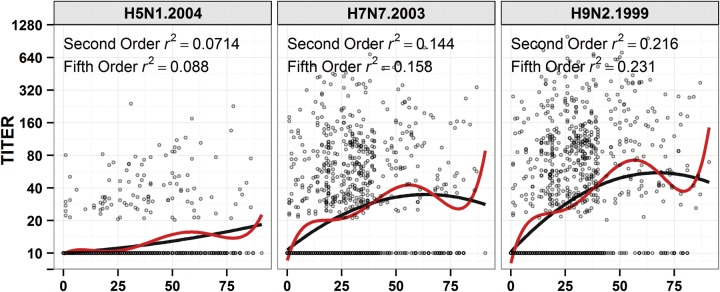
Scatterplot of avian strain titer against age with polynomial regressions lines. The black line denotes the second-order polynomial, and the red line denotes the fifth-order polynomial.

As noted in our article, the microarray assay used in this analysis is more sensitive than traditional HI and MN tests when comparing titers for the homologous antigens. A problem in doing studies to evaluate zoonotic exposure is that the exact antigenic composition of the infecting virus may be unknown. Therefore, HI/MN tests may yield false-negative results, a problem that is less evident with the microarray, which measures antibodies to the head of hemagglutinin and is, therefore, more broadly reactive within subtype. The differences we see in response between H9 and H5 avian strains (and to a lesser extent between H7 and H5 strains) are robust even when a much higher titer cutoff is used (up to 1:320, using microarray). With a cutoff titer of 1:20, 76% of individuals aged ≥50 years test positive for H9 antibodies (compared with 33% aged <50 years; χ^2^ = 89.9; *P* < .001). With a more conservative (and probably more appropriate) titer cutoff of 1:80, this percentage is 41% (18% among those aged <50 years; χ^2^ = 40.6; *P* < .001), suggesting that age distribution needs to be carefully taken into account when designing seroepidemiologic studies of avian influenza virus in humans. This result is robust for site effects in Vietnam, strengthening the hypothesis that this phenomenon is not related to poultry exposure.

Understanding the best way to interpret avian influenza virus serologic data, including cross-reactions generated by nonavian strains, is crucial for measuring incidence in both high-risk groups and the general population. The results generated by Pawar et al contribute to this understanding, but their study, as with all studies showing H9 positivity, should be interpreted with caution, as these H9-positive signals are possibly cross-reactions.

## Supplementary Data

Supplementary materials are available at *The Journal of Infectious Diseases* online (http://jid.oxfordjournals.org/). Supplementary materials consist of data provided by the author that are published to benefit the reader. The posted materials are not copyedited. The contents of all supplementary data are the sole responsibility of the authors. Questions or messages regarding errors should be addressed to the author.
